# Computational Studies of Snake Venom Toxins

**DOI:** 10.3390/toxins10010008

**Published:** 2017-12-22

**Authors:** Paola G. Ojeda, David Ramírez, Jans Alzate-Morales, Julio Caballero, Quentin Kaas, Wendy González

**Affiliations:** 1 Center for Bioinformatics and Molecular Simulations (CBSM), Universidad de Talca, 3460000 Talca, Chile; david.ramirez@uautonoma.cl (D.R.); jalzate@utalca.cl (J.A.-M.); jcaballero@utalca.cl (J.C.); 2 Facultad de Ciencias de la Salud, Instituto de Ciencias Biomedicas, Universidad Autonoma de Chile, 3460000 Talca, Chile; 3 Institute for Molecular Bioscience, The University of Queensland, Brisbane, Queensland 4072, Australia; q.kaas@imb.uq.edu.au; 4 Millennium Nucleus of Ion Channels-Associated Diseases (MiNICAD), Universidad de Talca, 3460000 Talca, Chile

**Keywords:** molecular dynamics simulations, databases, snake peptides, proteomics, molecular modeling

## Abstract

Most snake venom toxins are proteins, and participate to envenomation through a diverse array of bioactivities, such as bleeding, inflammation, and pain, cytotoxic, cardiotoxic or neurotoxic effects. The venom of a single snake species contains hundreds of toxins, and the venoms of the 725 species of venomous snakes represent a large pool of potentially bioactive proteins. Despite considerable discovery efforts, most of the snake venom toxins are still uncharacterized. Modern bioinformatics tools have been recently developed to mine snake venoms, helping focus experimental research on the most potentially interesting toxins. Some computational techniques predict toxin molecular targets, and the binding mode to these targets. This review gives an overview of current knowledge on the ~2200 sequences, and more than 400 three-dimensional structures of snake toxins deposited in public repositories, as well as of molecular modeling studies of the interaction between these toxins and their molecular targets. We also describe how modern bioinformatics have been used to study the snake venom protein phospholipase A2, the small basic myotoxin Crotamine, and the three-finger peptide Mambalgin.

## 1. Introduction

Snake venom is a complex mixture of proteins and peptides, and presents several medical and pharmaceutical applications [1,2,3]. Since the Greek antiquity, substances extracted from snake have been recognized for their medicinal properties, and the rod of Asclepius, a snake coiled around a staff, is the most commonly used symbol of medicine and health. In modern times, a number of notable molecules derived from snake toxins are used in the clinic or are in various stages of clinical development [4]. The most famous example of snake-derived medicine is captopril (Capoten), which was developed by Bristol-Myers Squibb, and is now used as a generic medicine for treating hypertension and congestive heart failure [5,6]. It is a small molecule inhibitor of the angiotensin converting enzyme (ACE), and is derived from bradykinin potentiating peptides found in the venom of the South American snake *Bothrops jararaca* [5]. Another snake-derived compound potentially used for heart failure is cenderitide (CD-NP, Mayo Clinic/Capricor Therapeutics, Beverly Hills, CA, USA) [7]. It is a chimera between the green mamba *Dendroaspis* Natriuretic Peptide DNP and the human C-type natriuretic peptides, and activates guanylyl cyclases. It completed Phase I/II clinical trial for chronic heart failures.

Eptifibatide (Integrilin, Millennium Pharmaceuticals/Merck, Cambridge, MA, USA), a RGD-motif cyclic heptapeptide that acts on glycoprotein IIb/IIIa integrin receptors of the blood platelets, is a medicine used in the clinic to prevent platelet aggregation and thrombus formation in acute coronary syndromes. It was derived from a much larger protein from the pygmy rattlesnake (*Sistrurus miliarius barbouri*). In the same category, anfibatide (Declotana, Lee’s Pharmaceutical, Hong Kong, China) is a snake venom-derived from platelet aggregation inhibitor peptide that antagonizes platelet glycoprotein Ib receptor. It is investigated for the treatment of acute thrombotic thrombocytopenic purpura, a fatal blood clot disorder [8]. It completed phase Ib–IIa, and will undergo phase II according to Lee’s website. It also has potential for treatment of acute experimental ischaemic stroke and reperfusion injury.

A “detoxified” variant of the α-cobratoxin (RPI-78M, ReceptoPharm/Nutri Pharm, Coral Springs, FL, USA), stimulates the production of cytokines. It has application in autoimmune diseases, myasthenia gravis (MG), muscular dystrophy (MD), and amyotrophic lateral sclerosis (ALS). It recently received, from the U.S. Food and Drug Administration (FDA), an orphan drug designation for the treatment of pediatric multiple sclerosis. Gyroxin is a serine protease from *Crotalus durissus terrificus*, and has applications aiming at tissue regeneration, repairing nervous system traumas and bone marrow. Phase I clinical trial found the protein to be safe and preclinical study showed promising results for treating chronic venous ulcers [9].

Counterintuitively, some snake toxins can inhibit pain, and have, therefore, potential application as analgesics. For example, crotalphine is a 14 amino acid peptide with one disulfide bond, and has analgesic properties through TRPA1 desensitization [10]. It was reported to be developed by the Brazilian firm Biolab Farmaceutica, but the compound is not mentioned on their website. The mambalgins are three-finger toxins with antagonist activity on Acid-sensing ion channel (ASIC) channels, and are active on a range of pain models [11]. They were to be developed by Theralpha, a French startup company, which has ceased activity.

The examples mentioned above stress why snake venom peptides attract scientific and medical interest [3], and considerable efforts are made to mine snake venoms for interesting new compounds. The venom of a single snake species (from the roughly 725 species of venomous snakes) contains hundreds of potentially pharmacologically active and useful molecules [12]. In recent years, important technological progress in transcriptomics and proteomics has resulted in a rapidly increasing knowledge of peptides and proteins in animal venoms, include those of snakes. These technologies enable rapid discovery of the nearly complete set of toxins of a snake venom [13,14,15], which was hitherto not achievable using previous methods based only on reversed phase-high performance liquid chromatography (RP-HPLC) and mass spectrometry [16]. The number of toxins unraveled by modern “omics” approaches is very large, efficient computational methods are required to mine this massive amount of data. This review will first focus on databases that allow searching and retrieving information on snake toxins. Computational approaches that have been developed to help study snake toxin activity, such as the prediction of toxin three-dimensional structures and of their interactions with molecular targets, will be described. The successes and challenges of predicting binding affinities and specificities using molecular modeling will be discussed, with the future perspective to integrate the biology of venoms and prediction of toxin biological targets. Finally, we will describe how modern bioinformatics have been used to study the activity and biological targets of the snake venom protein PLA2, the small basic myotoxin Crotamine and the three-finger peptide Mambalgin.

## 2. Extent of Our Knowledge on Snake Toxins

**Figure 1:**
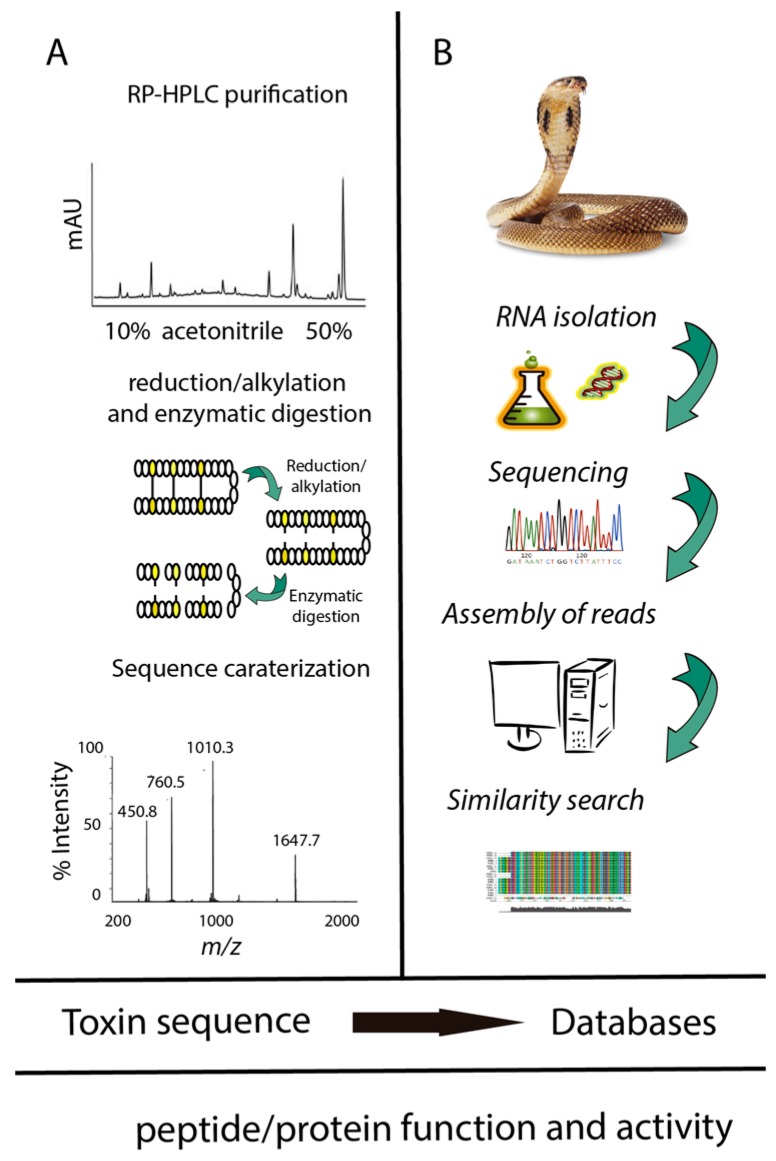
Integration of proteomics, transcriptomics, and databases in the study of venoms. (**A**) Classical peptidomics analysis of toxins; (**B**) nucleotide discovery from transcript to precursor sequence.

General databases, such as UniProt [17,18], NCBI Genbank/GenPept [19], and the Protein Data Bank (PDB) [20,21], play essential roles in simplifying access to information regarding protein sequences and three-dimensional structures [22]. However, the information about peptides and proteins from snake toxins is not standardized in these resources, especially the naming of toxins and pharmacological activities, and mining for snake venom peptides is difficult. Moreover, the database relies on depositions of information from authors to feed the data, leading to numerous duplications of entries, and a large body of work is being published in peer-reviewed articles, but never submitted to the general databases. Specialized databases, from venomous animals, are slowly emerging. Conoserver [23], Arachnoserver [24], and ISOB (Indigenous snake species of Bangladesh) [25] provide information on venoms from cone snail, spider, and snakes, respectively. A recently developed resource, VenomZone, is provided by the Swiss Institute of Bioinformatics (SIB), and has information about the venoms from six types of organisms, including snakes. Access to the information is divided in taxonomy, activity, and venom protein families, making it easy to search through the website. Furthermore, all the information is linked to the venom protein information from the UniProtKB/Swiss-Prot database (manually annotated and reviewed) and UniProtKB/Trembl (automatically annotated). The knowledge on the activity of peptides that have been fully characterized helps to predict the possible function of uncharacterized peptides and proteins. In this context, specialized databases play an essential role in providing access to data, predicting the three-dimensional structures and functions of toxins, and identifying outstanding toxins with potential new characteristics (Figure 1). However, there is presently no commonly established and standard way for practical annotation of toxins from the data sources mentioned above, leading sometimes to an erratic estimate for the number of toxins in the venom of one animal. Machine learning-based classifiers could help to solve this problem. ToxClassifier [26] is a machine learning tool that allows a consistent differentiation of toxins from non-toxin sequences, and reports the best-hit annotation, permitting assignment of a toxin into the most correct toxin protein family, providing increased curation of these existing databases [27].

### 2.1. Transcriptomic Analyses of Peptides and Proteins from Snake Venom Glands

Genome sequencing of venom gland is still highly expensive, and the assembly of genomic information and their analysis requires substantial bioinformatics commitment [28,29]. By contrast, sequencing cDNA libraries created from venom gland mRNA using next generation sequencing (NGS) and their assembly became mainstream in research. The transcriptomes of several snake venoms have been reported [30,31,32,33]. NGS platforms produce near comprehensive sequence transcript information coding for venom peptides and proteins, complementing the traditional PCR techniques, which could only discover toxin transcripts related to those already discovered in other species or from proteomic study. The most common NGS technologies used for venom transcriptomics, i.e., 454 GS FLX Titanium and Illumina, proceed by fragmenting the cDNA and sequencing all or part of these fragments [34]. The 454 technology, which is losing momentum and will be discontinued, produces longer stretch DNA sequence (reads) than the Illumina technology (limited to read of 150 bp), but of lower quality [34,35]. The fragments can be assembled back into full length or partial transcripts (the assembled fragments being called contigs) using different software, such as Trinity, Trans-Abyss or SoapdeNovo [36].

An older technology still in use today consists in using the classical Sanger sequencing of expressed sequence tags (ESTs), which are short DNA sequences obtained by sequencing the ends of transcript fragments. Given that most snake venom peptides are around 100 residues long, the sequencing of ESTs often yields the complete DNA sequence of a peptide. The EST approach has been successfully applied to venom from snakes, noting that the number of retrieved sequences are much smaller than generated by NGS, and typically only the most expressed transcripts will be identified using Sanger/ESTs [32,37]. The ESTs can be assembled back into the original contigs using several algorithms, such as CAP3 [38], Phrap [39], SeqMan [39,40], or MIRA [41].

Transcriptomic analysis generally provides insight into the peptide/protein profile of snake venom, and can be used for discovering putative new peptides and their isoforms [15,42,43,44,45], or peptides that are lowly expressed, and consequently hard to identify by proteomic analysis [46]. Finally, with advances in bioinformatics—which is no more than “the application of information science to biology” [47]—venom gland transcriptomic data is an excellent tool for studying peptides evolution [45,48], exploring antivenom and therapeutic agents [32,49], and understanding structure–function relationships [50].

### 2.2. Proteomic Analyses of Peptides and Proteins from Snake Venom Glands

Snake venom proteomes are highly complex mixtures of peptides and proteins [51]. Proteomic approaches to investigate snake venoms were recently reviewed [2,52]. These approaches generally use a combination of electrophoresis, liquid chromatography, Edman degradation sequencing, amino acid analysis, enzymatic digestion, and mass spectrometry, among other techniques. The methods more widely used are a combination of high performance liquid chromatography, Edman degradation, MALDI-TOF/MS of proteins, 1D or 2D PAGE and ESI/MS/MS sequencing of digested proteins [40]. The first step to study the proteomes of a snake venom is the venom extraction, which is performed by “milking” the living snake. Snake milking is achieved by forcing the snake to bite into a proper container. After venom collection, the proteins are separated using high performance liquid chromatography (RP-HPLC), ultra-high performance liquid chromatography (UHPLC), and exchange chromatography [53]. Once the crude venom is fractionated, the sequences of the peptides are determined using a combination of mass spectrometry and Edman degradation. Prior to the MS/MS analysis, the peptides are usually reduced, alkylated, and enzymatically digested (usually with trypsin or chemotryspin) [53]. Finally, the tertiary structure is studied using nuclear magnetic resonance spectroscopy [53,54].

**Figure 2:**
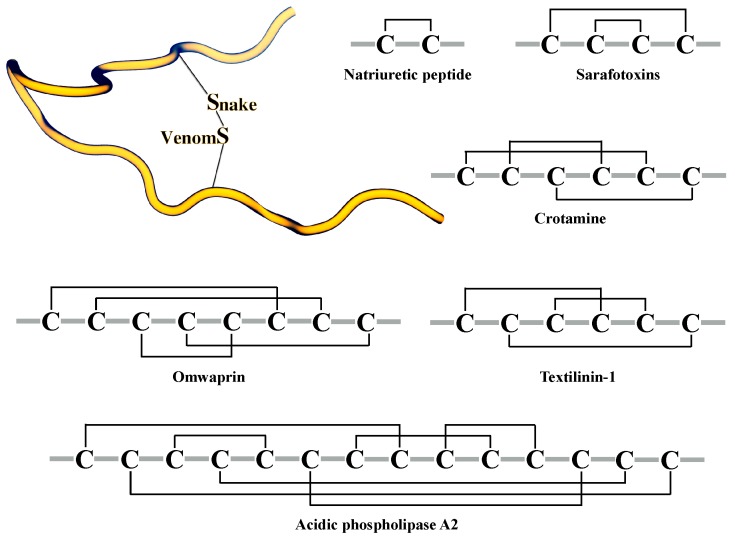
Cys patterns of some toxins from snake venoms. Cartoon representation of a disulfide bridge (top left). The disulfide bond arrangements are shown as black lines.

Peptides and proteins from snake venoms have a high content of cysteine residues in their primary sequences, and most of these cysteines form crosslinking disulfide bridges. The stabilization underpinned by the creation of disulfide bridges has been linked to several important features of snake toxins: enhancing activity, higher resistance to proteases, improving selectivity, and stabilizing secondary structure elements [55,56,57,58]. Furthermore, the number of disulfide bridges in snake peptides varies (Figure 2); for instance, natriuretic peptides present one disulfide bond, sarafotoxines present two disulfide bonds, and more complex toxins, such as omwarpin, have four disulfide bonds. In the widely-studied phospholipase A2 family, the acidic phospholipase A2 subfamily, with 142 proteins from snake venoms deposited in UniProt (search criteria: *taxonomy: “Serpentes (snakes) (8570)” protein “acidic phospholipase a2” (keyword: toxin OR annotation: (type: “tissue specificity” venom)) AND reviewed: yes*) share a characteristic disulfide bridge connectivity, where 14 Cys residues form seven disulfide bonds.

## 3. Snake Toxin Structures and Activities

To comprehend the structure and function of snake toxins will provide a better understanding of their role in venom toxicity. Elucidation of their structures will further help us to better understand the protein–protein interactions in snake venom, as well as their target receptor/ion channels [59]. Toxins in general seem to adopt a limited number of structural scaffolds. It was initially proposed in the 1970s that the 57 snake venom toxins described at the time as being neurotoxic or cytotoxic had similar secondary structure content [60]. The first snake venom 3D structure was also solved by X-ray diffraction in the 1970s (reported on 1978) [61]. It was released in the Protein DataBank (PDB) in 1981 under the PDB ID: 1NXB (snake venom curarimimetic neurotoxins) [61]. In the past 20 years, the discovery and unraveling of snake venoms has largely paralleled the technological development in proteomics and transcriptomic sciences. Additionally, the number of 3D structures has increased, due to the amazing progress of spectroscopic techniques, such as X-ray crystallography and nuclear magnetic resonance (NMR) spectroscopy [62]. Structural genomics appeared early 2000s, and had a dramatic influence on the structural study of snake venoms [63,64]. The increased pace at which 3D structure of snake toxins are deposited in the PDB in recent years is striking, with 101 and 409 3D structures deposited before and after 2000, respectively.

The specific structure for a given toxin is important to understand the molecular events at the origin of toxin activity. Based on these experimental structures, molecular modeling has been used to understand the molecular interactions related with toxin affinity and specificity.

### 3.1. Classification of Snake Venom Toxins

The majority of snake venom proteins, i.e., 2224 proteins and peptides, could be categorized into 30 families [65] (Table 1); whereas 12 proteins are not classified yet. To this day, 410 and 100 3D structures of snake toxins have been solved by X-ray crystallography or NMR spectroscopic techniques, respectively; and 37 3D structures have been modeled and reported in the Protein Model Portal of the PSI-Nature Structural Biology Knowledgebase [66]. This information was obtained from UniProtKB [67,68] by using the following search criteria: *taxonomy: “Serpentes (snakes) (8570)” (keyword: toxin OR annotation: (type: “tissue specificity” venom)) AND reviewed: yes*.

### 3.2. Structures of Snake Venom Toxins

There are several structural differences between snake venom families, starting with the size of the peptides: snake peptides can be classified as being very short (228 snake peptides are under 25 amino acids) and as being longer (1996 snake peptides and proteins are more than 26 amino acids). For instance, the bradykinin-potentiating peptide 7a snake venom from *Bothrops jararaca* has only seven amino acids, and the *Austrelaps superbus* venom factor 1 is the largest identified snake venom proteins, with 1652 amino acids). A second structural feature used to easily classify snake toxins is the presence of disulfide bridges. Disulfide bonds confer rigidity, stability, and resistance to denaturation, but also give the molecule some flexible domains that are important for target recognition, and more recently, for engineering purposes [69,70].

There are several toxins folds, and they can be classified according to the ion channel they are active on, or the type of fold resulting after peptide oxidation [71]. The peptides and proteins found in snake venoms with high content of disulfide bonds and the different resulting frameworks, structures, and biological functions, were recently reviewed by Reeks et al. [1].

#### 3.2.1. ICK Fold

**Figure 3:**
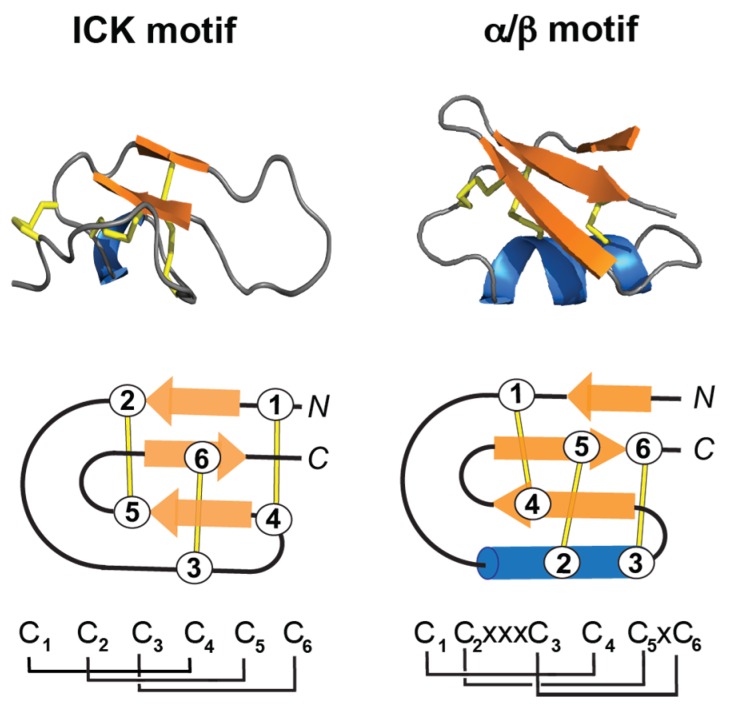
3D structure of the inhibitor cystine knot (ICK) and the CSα/β motif. The disulfide bonds are shown in stick format or lines (yellow), the β-sheet in arrow format (orange), and the α-helix in blue. (**Top**) 3D structure of maurocalcin (PDB: 1C6W) and charybdotoxin (PDB: 2CRD). (**Bottom**) Schematic representation of the ICK motif, and the CSα/β motif. The disulfide bond connectivities for each motif are shown at the bottom of the panel, where “C” means cysteine and “x” shows the conserved spacing between cysteine residues. The cysteine residues are labeled 1 to 6.

The inhibitor cysteine knot (ICK) motif is a structural fold displayed by a large number of peptides with diverse sequences, length, and activities, and present in all kingdom of life [72]. The ICK contains a ring made by two disulfide bonds (Cys I-IV, Cys II-V), the third disulfide bond (Cys III-VI) penetrates the ring to form the “knot” (Figure 3) [73]. Peptides containing ICK motif are 26–50 residues long, and present different activities, including ion channel blockers, hemolytic, antiviral, and antibacterial peptides [74]. ICK peptides are also very stable to chemical, thermal, and biological denaturation. Several reviews describe, in detail, their structural characteristics and biomedical applications [71,73,74,75,76].

#### 3.2.2. α/β Fold

A structural motif also found among toxins present in snake venoms is the CSα/β motif (cysteine-stabilized α/β) (Figure 3). The CSα/β motif is composed of an α-helix and an antiparallel triple-stranded β-sheet stabilized by three or four disulfide bonds [77]. Peptides containing the CSα/β motif are more abundant in scorpions, and include sodium, potassium and chloride channels modulators [78]. Crotamine has the overall fold of a prototypical alpha/beta toxin, and it will be described in Section 3.4.

### 3.3. Molecular Modeling of Snake Toxin Structures

Molecular modeling of snake toxins aims at providing atomistic explanations of their biological activity in terms of structure, dynamics, and molecular interactions. Structure-based molecular modeling methods, such as docking and molecular dynamics (MD) simulations, require a 3D structure of the toxin as a starting point. The 3D structures of 510 snake toxins have been solved by X-ray crystallography and NMR spectroscopy. These structures serve as templates to build homology models from structurally uncharacterized snake toxins. In the absence of an experimentally resolved structure, this technique can give a 3D model for a toxin that is evolutionary linked to at least one identified protein structure. Homology modeling predicts, then, the 3D structure of a certain toxin sequence (target) based on its alignment to one or more proteins of known structures (templates) [79]. Most homology models have been built using software such as Modeller [80]. Other programs used to perform homology models are ICM [81], module Prime in Schödinger suite [82], as well as web servers such as SWISS-Model [83] and I-TASSER [84]. Using the force field that has been given to the atoms in the system, it is possible to find a stable conformation or a minimum on the potential energy surface in order to start MD. There will be more than one local minimum for a toxin. In principle there may be a global minimum, but this will not likely be found without an extensive conformational search. The initial energy minimized structure is usually subjected to molecular dynamics to study the motion of molecules with respect to time. MD represents an option to study the structure and dynamics of snake toxins at atomistic resolution simultaneously. The growing significance of MD simulations for structural prediction has been highlighted by the critical assessment of structure prediction (CASP) experiments, where MD turned out to improve the model refinement notably [85].

MD simulation [86] is based on the numerical integration of the classical Newtonian equations of motions for all the atoms in a system. The interactions between atoms are described by physic-based force fields, such as AMBER [7], CHARMM [8], Gromos [9], and OPLS [10], among others. The force fields have been fitted to reproduce values from experiments or gas phase quantum mechanical calculations [87]. Short MD simulations are frequently employed to refine the conformation of homology models of snake toxins. MD simulations are also employed to suggest the molecular interaction between toxins and their target, and for the rational design of novel inhibitors using an initial pose, often resulting from docking [88,89].

### 3.4. Molecular Modeling of Snake Toxin—Target Complexes

**Figure 4:**
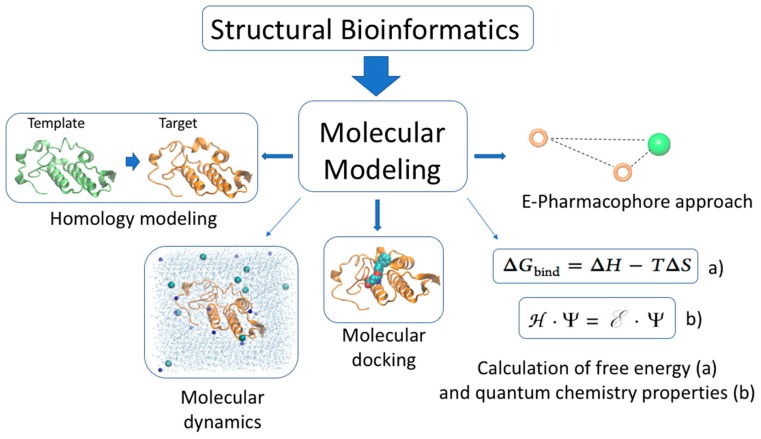
Molecular modeling is a branch of structural bioinformatics. Molecular modeling comprises several methods to simulate the behavior of molecules and molecular systems. Some of them, such as homology modeling, molecular dynamics, molecular docking, free energy, quantum chemical calculation, and e-pharmacophore approach, have been used to study the activity of snake venom proteins.

Molecular modeling can provide structural information and theoretical understanding that is not easily derivable from experimental results. Molecular modeling comprises the ways to simulate the behavior of molecules and molecular systems. Nowadays, this definition is ever associated with computer modeling [90], and in consequence, is a branch of the structural bioinformatics. Various molecular modeling techniques (Figure 4) [91,92] have been used to understand the molecular interactions at the origin of toxin affinity and specificity. Docking approaches use heuristic algorithm to produce a large number of docked “poses”, which are then clustered and ranked using knowledge from experiments, or on the basis of a scoring function. Despite that they have given valuable insights about protein-ligand binding modes, docking methods are not reliable for predicting binding energies, due to the simple scoring functions they use [93]. An effort to improve affinity prediction with docking is typically performed using a rescoring process with other simple functions or solvated-based scoring functions. The poses generated by the docking program are taken, and methods such as MM/PBSA (molecular mechanics/Poisson–Boltzmann surface area) or MM/GBSA (molecular mechanics/generalized Born surface area) [64,94,95,96,97] can be used to improve docking accuracy [98]. Another strategy is the use of MD simulations to sample the conformations of the complexes obtained using docking, and subsequent calculation of the binding energy by averaging the score values for different poses extracted from the trajectory [99,100]. Under this approach, the receptor flexibility and the presence of water molecules contribute to a more realistic description of the complex, which could have an influence in binding energy calculations.

The molecular foundation of the bioactivity of most snake toxins relies on the recognition by an interface ligand region toward the complementary surface of the receptor. On the ligand side, the atoms involved in the interaction are usually defined as the pharmacophore. When chemical knowledge of numerous active toxins in a receptor is available, one can detect a common pharmacophore between them. A novel method to develop energetically optimized, structure-based pharmacophores for use in rapid in silico screening to detect similar ligands (potentially active) was developed by Salam et al. [101]. This approach has been used to identify potential specific inhibitors of snake proteins, such as PLA2.

The present major bottleneck in snake toxin investigation is the determination of the activity of individual toxins, and several molecular modeling approaches could potentially help to solve this problem. In the following section, we will describe how homology modeling, molecular dynamics, molecular docking, free energy, quantum chemical calculation, and e-pharmacophore approach have (Figure 4) been used to study the activity of the snake venom protein PLA2, the small basic myotoxin crotamine and the three-finger peptide mambalgin.

### 3.5. Molecular Modeling Applied to the Study of PLA2, Crotamine and Mambalgin

#### 3.5.1. PLA2

Phospholipases A_2_ (PLA2; EC 3.1.1.4) are proteins present in snake venoms with a digestive role in phospholipid hydrolysis [102]. They specifically hydrolyze the sn-2 ester bond of phospholipids, releasing fatty acids from the second carbon group of glycerol, and display enhanced catalytic activity in micellar and lamellar aggregates, both in membranes and at other lipid–water interfaces [103]. When a snakebite occurs, PLA2 toxins exhibit a wide variety of pharmacological effects on the normal physiological processes of victims, such as myotoxicity, neurotoxicity, and edema-inducing activity [104,105]. Due to their toxic pathophysiological role, there is a considerable pharmacological interest towards the design and discovery of PLA2 specific inhibitors for antivenom therapies in humans. 

There are several reports where computational molecular modeling methods have been used for characterizing some functional aspects of PLA2s, or the development of PLA2 inhibitors that contribute to the weakening or annihilation of snake venom toxicity. These applications use the X-ray crystallographic 3D structural information generated in the last decades, and methods such as molecular dynamics (MD) simulations and docking.

**Figure 5:**
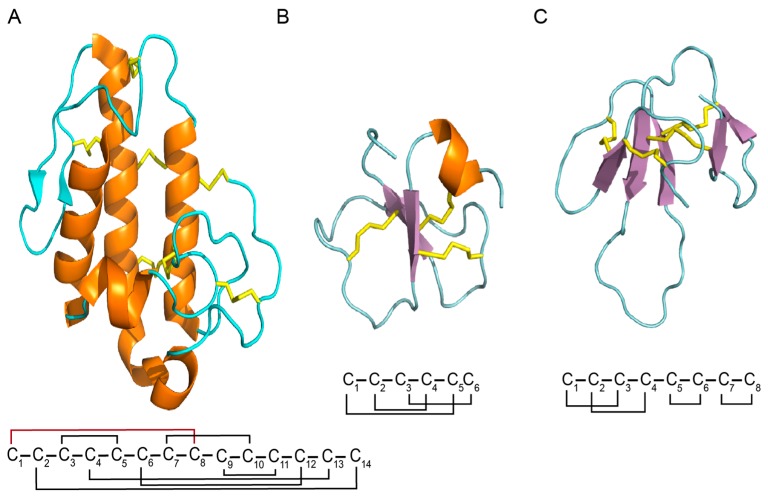
Cartoon structure of snake toxins. (**A**) Structure of PLA2 (PDB ID: 1PPA); (**B**) structure of crotamine (PDB ID 15HO); (**C**) structure of mambalgin 2 (PDB ID: 2MFA). Disulfide bond connectivities are shown as yellow sticks, the β-sheets are shown as arrows, and the α-helices are shown in orange. The disulfide bond connectivity shown in red for PLA2 represents the difference between class I and II.

Structural architecture of snake venom PLA2s is divided into classes I and II, based on their amino acid sequence and disulfide bonding pattern [106]. However, they have a conserved structure which contains an N-terminal α-helix (H1), a Ca^2+^-binding loop, two antiparallel α-helices (H2 and H3), a two-stranded antiparallel sheet (β-wing), and a long C-terminal loop. In general, folding is stabilized by seven disulfide bonds (with different pattern in classes I and II) (Figure 5A). Some PLA2s undergo aggregation in a concentration-dependent manner. Crystal structures available for several PLA2s confirm that they can form associations in dimer, and more units with physiological implications.

**Figure 6:**
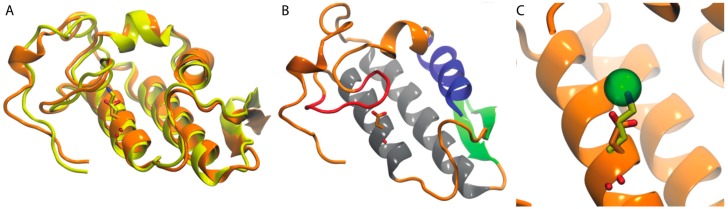
Acidic and basic PLA2s share a common molecular architecture. (**A**) Homology models of the acidic PLA2 AplTx-I (orange) and the basic PLA2 CoaTx-II (yellow). Their different amino acids at position 49 are shown in licorice; (**B**) the architecture of PLA2s is shown, using for this purpose, the homology model of AplTx-I. N-terminal α-helix (blue), Ca^2+^-binding loop (red), antiparallel α-helices (gray), two-stranded antiparallel-sheet (green); (**C**) The analysis of position 49 at AplTx-I and CoaTx-II, superimposing the structures with the monomeric PLA2 from *Agkistrodon halys pallas* (PDB ID: 1M8R) show that Nε atom of K49 occupies the position of Ca^2+^ in the catalytically active Asp49 PLA2. Cadmium ion which in 1M8R occupies the site of Ca^2+^ is shown in licorice.

The conserved residue, Asp49, plays a key role coordinating Ca^2+^ in the catalytic site of PLA2s, thus assisting the stabilization of the intermediary transition state in catalysis; however, there are PLA2 homologues in which Asp49 is changed to lysine. These K49 PLA2s are catalytically inactive, but retain cytolytic activity, and destroy the integrity of synthetic liposome membranes by a Ca^2+^-independent process. Crystal structures of K49 PLA2s reveal that the Nε atom of K49 occupies the position of Ca^2+^ in the catalytically active Asp49 PLA2s [107]. Recently, our group published two homology models, their respective careful model validation, and molecular dynamics simulations of a) one Asp49 PLA2 purified from *Agkistrodon piscivorus leucostoma* snake venom (AplTx-I) and b) a K49 PLA2 (CoaTx-II) purified from *Crotalus oreganus abyssus* (Figure 6A). AplTx-I and CoaTx-II exhibit an expected common molecular architecture and secondary structure similar to that of other PLA2s (Figure 6B), except the residue 49 (Figure 6C).

The majority of molecular modeling applications in literature for studying PLA2s are oriented to rational design of novel inhibitors for the treatment of different *Viperidae* snakebites. Some examples are cited here:

Most examples have been applied to PLA2 of *Daboia russelii*. Recently, Nargotra et al. [108] evaluated a library of natural products and synthetic molecules through docking studies on *D. russelii* PLA2 to identify possible inhibitors. Their study lead to in silico identification of several molecules as PLA2 inhibitors, with most of them belonging to phenolic and substituted benzaldehydic compounds. It is important to note that the selection in this work was performed by considering docking energy scores, which is a reliable criterion, according to literature [60]. The same authors proposed the docking poses inside PLA2 of *D. russelii* for synthetic phenolic compounds effective against snake venom [109]. They found that phenolic compounds having hydroxyl and methoxyl groups in their benzene ring showed maximum inhibitory potency, as they form hydrogen bonds with the residues Asp49 and Gly30 in the binding site of *D. russelii* PLA2 (these residues configurate together with Gly30 and Tyr28 a Ca^2+^ coordination site and are involved in the binding of several ligands reported in Protein Data Bank).

Other works applied to *D. russelii* PLA2 are listed below: Anilkumar et al. [110] docked imidazopyridine derivatives inside the binding site of *D. russelii* PLA2, and they found that compounds form π–π stacking interactions with Trp31, and extend towards Gly32, potentially adding further amide–π stacking contributions. Yadava et al. [111] docked nine pyrazolo[3,4-d]pyrimidines inside *D. russelii* PLA2 for describing their binding modes. They found that the studied compounds have better docking binding energies than indomethacin, however, it is necessary to prevent, again, the use of docking binding energies for comparing the affinities of two molecules [93]. In other work, Ramakrishnan et al. [112] generated pharmacophore models based on the interaction of different types of inhibitors (peptides, vitamin E, indole derivatives, and nonsteroidal anti-inflammatory drugs) with their preferred subsites in the active site of the subunit A of *D. russelli* PLA2. Authors validated the final model and subjected it for screening a library of drug-like compounds. They identified eight compounds and subjected them to molecular docking and MD simulation, to assess their binding mode with both subunits. After analyzing these computational experiments, they selected four compounds for further biochemical assays, and found that two compounds can bind both the subunits of PLA2 of *D. russelli* venom, in spite of its aggregated form. Sivaramakrishnan et al. [113] reported an integrated approach involving homology modeling, MD, and molecular docking studies on *D. russelli* venom PLA2 fraction V belonging to Group IIB secretory PLA2 from *D. russelli*, in order to study the structure-based inhibitor design (3D structure of *D. russelli* PLA2 fraction IIIa was used as template, with >93% of identity). Authors also constructed a pharmacophore model, and identified potential specific inhibitors. Additionally, they highlighted the role of His47 and Asp48 within the PLA2 binding pocket as key residues for HB interactions with ligands.

Finally, Ramakrishnan et al. [114] performed comparative MD simulations of free and inhibitor-bound form of secretory *D. russelli* PLA2. This enzyme dimerizes asymmetrically with different orientation of Trp31 at the gateway of the active site of both the subunits A and B. Hence, the active site of subunit A is open, and that of subunit B is inaccessible to monodispersed inhibitors. Authors performed MD simulations for monomer and dimer forms of PLA2s in both native and complex forms (the bovine pancreatic PLA2 was selected as the monomeric form). They reported a comparison of trajectories with respect to fluctuation and deviation, which discloses the dynamics of surface and calcium-binding loops, as well as the difference in dynamics of active site residues. Their study discloses the sort of restrictions in *D. russelli* PLA2 active site for inhibitor binding, and implies suitable sites for further design of inhibitors based on active site scaffold.

Other recent works were applied to study the interactions between drugs and other PLA2s with X-ray structures available in PDB. For instance, in a recent work, Pereañez et al. [115] studied the mode of action of morelloflavone with PLA2 of *Crotalus durissus terrificus*, using docking. Authors found that morelloflavone occupies part of the substrate binding cleft of *C. durissus* PLA2, forming hydrogen bonds (HBs) with the residues Gly33, Asp49, Gly53, and Thr68 of the enzyme, and π–π stacking with the residue Tyr52. The same authors used docking to investigate the interactions between *C. durissus* PLA2 and bile acids, such as cholic acid (CA) and ursodeoxycholic acid (UDCA) [115]. Authors found that bile acids interact with the binding active site of PLA2 through different interactions, CA showed HBs with His48, whereas, UDCA showed HBs with Asp49 and Tyr28. In other work, Zhang et al. [116] docked structural elements of the persimmon tannin PT40 (a highly galloylated condensed tannin with an unusual flavonol terminal unit) inside Chinese cobra (*Naja atra*) PLA2 binding site, to understand the inhibitory mechanism of this natural product. They found that the residues Trp18, Try27, Gly29, His47, and Tyr63 are involved in the interactions. Finally, Chavan and Deobagkar [117] applied docking and MD simulation techniques to propose the putative interactions of LT10 peptide (small synthetic peptide derived from N-terminal of the lethal toxin neutralizing factor) with *Naja naja* PLA2. MD was performed to analyze the stability of the complex obtained by docking method.

Other applications used available structures in PDB for creating comparative models. For instance, Chinnasamy et al. [98] modeled the 3D structure of PLA2 of *Naja sputatrix* (Malayan spitting cobra) using the structure of *N. naja* PLA2 as template, applied 10 ns MD to the get stable conformations of the studied protein, and used the final structure to perform high throughput virtual screening by performing massive docking of compounds from different databases. After applying this protocol, authors selected seven compounds based on the docking score and free energy binding calculations. In other work, Chavanayarn et al. [118] studied the binding of the antibodies V_H_H-P3-1, V_H_H-P3-3, and VH P3-7 to PLA2 of *Naja kaouthia* (monocled cobra), using docking methods. They developed a homology model of the *N. kaouthia* PLA2 using *N. atra* PLA2 as template, and found that the antibodies covered the areas around the PLA2 catalytic groove and inserted their complementarity determining regions (CDRs) into the enzymatic cleft. Finally, Hage-Melim et al. [119] constructed a homology model of PLA2 of *Bothrops jararacussu* using a survey of complexes of PLA2 deposited in PDB. Authors carried out the pairwise alignment through involving eight sequences selected by crystallographic criteria, followed by a multiple alignment with the sequence of *B. jararacussu* PLA2. Authors claimed that X-ray structures of *B. jararacussu* PLA2 are in PDB, but no structure in complex with any inhibitor is available. Therefore, they performed the homology modeling to get a correct description of the binding site. They performed virtual screening in a large database, yielding a set of potential bioactive inhibitors, and confirmed the important role of Lys49 for binding ligands.

Fewer applications have been focused to study functional characteristics of PLA2s; however, there are some reports with interesting, more specific purposes. For instance, Murakami et al. [120] performed MD simulations of bothropstoxin-I (a K49 PLA2 of *Bothrops jararacuss* with myotoxic and neurotoxic activities) to study its complex with suramin (a polysulphonated naphthyl urea derivative). Instead, another report uses molecular modeling for studying the interactions between one PLA2 and one lipid. In this report, Abiram and Kolandaivel [91] studied the interaction of myristic fatty acid with acutohaemolysin and piratoxin-II (K49 PLA2s from *Bothrops pirajai* and *Agkistrodon acutus* respectively) using the hybrid two layered ONIOM (B3LYP/6-31G*: UFF) method [121]. Specifically, authors performed quantum chemical calculations on the tripeptides AFA and AVA present in acutohaemolysin and piratoxin-II. They found that the mode of interaction of the fatty acid with protein is electrostatic, confirmed further through molecular electrostatic potential maps, and the AFA shows stronger interaction than AVA, validating the impact of mutation on catalytic activity. The preferred secondary structural configuration and conformational properties of AVA and AFA validated the strong interaction of fatty acid with phenylalanine.

Other report tried to explain the higher activity of PLA2s at solvent–lipid interface. In this report, De Oliveira et al. [122] performed MD simulations of PLA2 of *Agkistrodon halys pallas* in water, methanol, and octanol. Authors used these simulations to propose an interfacial activation model for PLA2 in atomic detail. When the enzyme is in a more hydrophobic environment, they noted that a series of conformational changes occurs: (a) increase of solvent accessible surface area; (b) side chain reorientation of Asp49 residue that allows Ca^2+^ coordination; (c) reduction of the distance between His48 and Asp49, increasing the nucleophilicity of Nε-His48; (d) reorganization of calcium binding loop; (e) side chain reorientation of Trp31, which defines a new specificity pocket for the phospholipid chain; and (f) a reorientation of Lys69 side chain, allowing access to the active site. These findings are related with biochemical and structural studies, and provide information concerning the process of interfacial action in PLA2.

As a last example, the following report used molecular modeling for explaining the differences between functional properties of different PLA2s present in the same organism. In the referred work, Vieira et al. [123] investigated the protein named Intercro (IC), a PLA2 present in the *Crotalus durissus terrificus* (South American rattlesnake) venom. They described, for the first time, the biochemistry of IC, and performed functional and structural studies to compare this molecule with other PLA2 proteins present in *C. durissus terrificus*. Authors developed a homology model of IC using crotoxin B, the basic PLA2 from *C. durissus terrificus*, as template; after this, they subjected the model to MD simulations in the presence of explicit water molecules to relax the system. They found that IC displays significant similarities in 3D structure with respect to crotoxin B. IC keeps an enzymatic activity similar to the crotoxin B isoforms (there are three isoforms structurally solved to date: CBa_2_, CBb, and CBc); however, it shows low myotoxicity and a total absence of neurotoxicity, indicating that IC presents a distinct pattern of biological activity. Authors used structural information observed in the IC model to explain an additional point [123]. It is known that the efficiency of the crotoxin complex for producing neurotoxic effect depends on the ability of crotoxin A to drive crotoxin B to the nerve terminal; therefore, they hypothesized that the interaction between IC and crotoxin A maybe does not exist, or is not able to drive IC to the nerve terminal. There are highly conserved residues in the N-terminal α-helix H1, active site region, Ca^2+^ binding loop, β-wing, and α-helix H3; however, they noted that there are 11 variable positions between the amino acid sequences of IC and those of the crotoxin B isoforms CBa_2_, CBb, and CBc. An inspection of the IC model revealed that all these positions correspond to amino acid residues placed on the IC surface. In this regard, the IC amino acid residues Phe70, Leu117, and Phe120 are exclusively present in the IC sequence, whereas the same positions in the isoforms CBa_2_, CBb, and CBc are occupied by the residues Trp70, Tyr117, and Tyr120. Previous literature supports that His1 and Trp70 of crotoxin B isoforms (His1 is only in CBb and CBc) are key residues involved in the formation of the heterodimer between crotoxins A and B [124]; based on this, Vieira et al. infer that the absence of a neurotoxic active crotoxin A/IC complex may be attributed to the presence of IC mutations (His for Ser at position 1, and Trp for Phe at position 70), which probably impairs the formation of a stable crotoxin A/IC interface. This example illustrates how the modeling of PLA2 structures and a sound analysis of previous literature could contribute to explaining a finding related to the biochemistry of the protein under investigation.

#### 3.5.2. Crotamine

Several proteins that are contained in snake venoms are responsible for their neurotoxic, cardiotoxic, hemorrhagic, and myotoxic activities. Among these, crotamine, which is a small basic myotoxin, binds strongly to excitable membranes, leading to the contraction of skeletal muscles [125,126]. Isoforms of crotamine (F2 and F3) were isolated from the venom of the South American rattlesnake *Crotalus durissus terrificus* by a single step of RP-HPLC [127]. It is a basic, low-molecular weight toxin, with a molecular mass of 4.5–5.0 kDa [128]. From 42 amino acid residues in crotamine, six are cysteines that form three disulfide bonds (Figure 5B) [129,130]. Crotamine acts on the voltage sensitive Na^+^ channels of the skeletal muscle sarcolemma, inducing a sodium influx by the opening of the ryanodine receptor. The toxin also seems to alter the Ca^2+^ ion influx in the sarcoplasmic reticulum [131]. Thereby, it is accepted that its general biological action is the depolarization of cell membranes. For a deeper understanding of crotamine isolation, structural and functional characterizations, as well as its potential biotechnological and therapeutic values, please see the reports published by Oguiura et al. [132] and Kerkis et al. [133].

Crotamine was also characterized as a cell-penetrating protein (CPP) with nuclear localization in vitro and in vivo [134]. Many biologically active compounds, including macromolecules, that are used as various kinds of drugs, must be delivered to the interior of cell or organelles, such as mitochondria or nuclei, to achieve a therapeutic effect. CPPs are a new means for transporting of macromolecules through the cell membrane that became relevant in the last years. For an extensive review on the topic, please see work published by Ruczynski et al. [135].

The use of computational methods to study crotamine is scarce, however, some computational techniques have been used to obtain and characterize its structure. For instance, Siqueira et al. proposed a theoretical 3D model for crotamine. They started from a homology modeling procedure, followed by intensive molecular dynamics (MD) simulations in water and complementary CD experiments. As no tridimensional structure of crotamine was available at that moment, the reported model was the first example for the 3D structure of this family of small basic myotoxins [136]. From this work, they proposed that key residues could be found in what they called the L1, L2, and L3 loops, which could serve in functions such as membrane anchoring, receptor anchoring, receptor isoform selection, and receptor inactivation. Later, Nicastro et al. reported the crotamine solution structure determined by proton NMR spectroscopy. A comparison of determined crotamine structure with human β-defensins showed a similar fold and a comparable net positive potential surface [137]. Moreover, the presence of the α/β scaffold and the existence of a surface characterized by a positive electrostatic potential seemed to justify the functional similarity with the Na^+^ channel affecting scorpion α-toxins. According to the authors, the most significant difference between the theoretical 3D model reported by Sequeira et al. and the NMR-derived structures from their work was the lack of a N-terminal α-helix segment. One reason for that could be the choice of the bovine β-defensin, BNBD12 [138], as a template. A new, and more refined, NMR structure determination of crotamine in aqueous solution at pH 5.8 and 20 °C, using standard homonuclear 1H NMR spectroscopy at 900 MHz, and the automated structure calculation software ATNOS/CANDID/DYANA was reported by Fadel et al. [139]. According to their results, the core of the protein is formed by an antiparallel β-sheet composed by residues 9–13 and 34–38. A long, non-regular loop connects the two strands of the β-sheet. The disulfide bridges connect the β-sheet to the N-terminal α-helix (Cys4/Cys36) and to this loop (Cys11/Cys30 and Cys18/Cys37). All nine lysines, with the sole exception of Lys35, and the two arginines, are oriented toward the solvent, so that crotamine has extended positively charged molecular surface areas. The global fold and the cysteine-pairing pattern of crotamine were similar to the β-defensin fold [138], although the two proteins have low sequence homology, and displayed different biological activities. Moreover, a generally applicable new computational protocol was introduced to determine unknown disulfide bond connectivity in globular proteins.

Starting from the hypothesis that reciprocal relationships existed between antimicrobial and cytotoxic host defense peptides, Yount et al. compared in phylogeny, 3D structure, target cell specificity and mechanisms of action of the human antimicrobial peptide hBD-2 and rattlesnake venom toxin crotamine [140]. Computational molecular docking was used to compare hBD-2 versus crotamine intermolecular interactions with prototypic bacterial, fungal, or mammalian Kv channels, based on the well-known fact that crotamine targets eukaryotic ion channels. The channel–toxin docking models supported direct interactions of each peptide with Kv channels. However, while crotamine localized to occlude Kv channels in eukaryotic, but not prokaryotic cells, hBD-2 interacted with prokaryotic and eukaryotic Kv channels, but did not occlude either. The authors stated that these insights might accelerate development of anti-infective or therapeutic peptides that selectively target microbial or abnormal host cells.

More recently, a theoretical study, based on semi empirical, ab initio and density functional theory (DFT) quantum methods, was performed to investigate the structural properties of two crotamines isolated from the venom of *Crotalus durissus*. Two protein models (I-a and II-a), representing crotamine fragments (I17-C18-L19-P20-P21) and (I17-C18-I19-P20-P21) respectively, were minimized, and their chemical properties (atomic charge, orbital population, and MO energy) were calculated to study differences in their myonecrotic activity. The authors concluded that even though there were some variations in the chemical properties between both fragments, no rigorous relationship to their respective biological activities could be established [92].

#### 3.5.3. Mambalgin-1 and -2

Mambalgin-1 and mambalgin-2 were identified as a new class of three-finger peptides from the venom of black mamba snake [141]. According to authors, these peptides were able to abolish pain through inhibition of acid-sensing ion channels (ASICs) expressed either in central or peripheral neurons. Mambalgins were not toxic in mice, but showed a potent analgesic effect upon central and peripheral injection that was as strong as morphine. The three-dimensional structure of mambalgin-1 was modeled from five templates of three-finger snake toxins through software Modeller 9 (Version 8, Andrej Sali, San Francisco, CA, USA, 2010) [80,142]. They were composed of 57 amino acids with eight cysteine residues, and only differed by one residue at position 4. The model structure presented a concave face commonly found in neurotoxins, and was stabilized by four disulfide bonds, with a pattern identical to that observed in the crystal structure template (Cys1–Cys3, Cys2–Cys4, Cys5–Cys6, and Cys7–Cys8) (Figure 5C). Mambalgins showed a strong positive electrostatic potential, calculated with the adaptive Poisson–Boltzmann solver [143], that may contribute to binding to negatively charged ASIC channels. The authors concluded that their findings identified new potential therapeutic targets for pain, and introduced natural peptides that could block them to produce a potent analgesia [141]. Later, same authors combined bioinformatic and functional approaches to uncover the molecular mechanism of channel inhibition by the mambalgin-2 pain-relieving peptide.

They first used homology modeling to obtain the structural models of rASIC1a and rASIC2a that were generated based on the structures of cASIC1a (57–90% sequence identity) using Modeller 9v8 [80], to obtain the homology models of the open and desensitized forms of the trimer complex based on the experimental structures (Protein Data Bank codes 4FZ0 and 3HGC, respectively). The three-dimensional structure of mambalgin-2 was obtained from experimental Protein Data Bank file 2MFA [144]. Afterwards, molecular docking experiments were performed to model the toxin-channel interactions. In silico rigid body docking of the toxin model onto the homology models of rat ASIC1a and ASIC2a were performed using the protein–protein docking program ZDOCK (version 2.3.2f, ZLAB, Chicopee, MA, USA, 2003) [145]. From those findings, the authors proposed a model where mambalgin-2 traps the channel in a closed conformation by precluding the conformational change of the palm and β-ball domains that follows proton activation. These data could help to understand inhibition by mambalgins, and provided clues for the development of new optimized blockers of ASIC channels [146]. Almost at the same time, Schroeder et al. [144] demonstrated the efficient chemical synthesis of the analgesic venom peptide mambalgin-2. To do so, they used a combination of solid-phase peptide synthesis and native chemical ligation. Then, using homonuclear NMR, the authors determined the structure of the synthetic toxin, and moreover, they revealed an unusual three-finger toxin fold reminiscent of functionally unrelated snake toxins. Furthermore, their functional data suggested that the mambalgins bind near the acidic pocket of ASIC channels in a manner very similar to that of PcTx1, most likely by insertion of one of their protruding “fingers”. Recently, in the same spirit of abovementioned researches, Mourier et al. [147] published the first full stepwise solid phase peptide synthesis of mambalgin-1, reported the determination of its three-dimensional crystal structure, and confirmed the biological activity of the synthetic toxin both in vitro and in vivo. Also, they used molecular docking experiments with ZDOCK [145] to carry out the protein–protein docking simulations of rASIC1a to mambalgin-1 crystal structures. Considering alanine scanning data, double mutant analysis, and X-ray structures, they generated new toxin-channel binding mode predictions by using in silico rigid body docking of toxin crystal structures onto the homology model of rat ASIC1a channel. In that way, the functional domain of the toxin for ASIC1a inhibition was delineated, supporting a crucial role of loop II (more precisely, in the face containing Phe-27, Leu-32, and Leu-34 residues) in the toxin–channel interaction. Finally, the proximity of mambalgin-1 Leu32 residue with Phe350 residue in rASIC1a, suggested by double mutant cycle experiments and the localization of critical toxin interacting residues, were exploited to propose a structural model of the toxin-channel complex. 

Altogether, the discussed data suggest that structural study of protein–toxin interactions is very relevant in biotechnological and medical fields, for instance, in the search for novel drug leads for the treatment of diseases involving ion channels or antimicrobials, as has been pointed out recently by Zhang et al. [148] and de Oliveira et al. [149], respectively. However, the molecular modeling approaches, such as molecular docking, homology modeling, electronic structure methods, and MD simulations, in which the interactions between toxins, like crotamine and mambalgins, and proteins (e.g., ion channels) can be examined in atomic detail, have been scarcely used in the field. This can offer us many possibilities, from computational simulations to exploit the available structural data for these proteins, and other toxins from snake venoms, with the aim to find new medical or biological applications.

### 3.6. Conclusions

Snake toxins have been instrumental in developing new medicines, and are actively pursued as drug leads [4]. The venoms of snakes represent a large library of active compounds, and we have shown here how modern computational biology and chemistry are used in many aspects of their initial characterization, from the discovery of genes and proteins to the determination of their three-dimensional structure and interaction with molecular targets. In this review, we particularly focused on the molecular modeling studies of PLA2, crotamine, and mambalgin, which were chosen as representative of the breadth of current computational techniques, but other important snake compounds have been studied by molecular modeling, e.g., α-bungarotoxin and other three-finger toxins targeting the nicotinic acetylcholine receptors [150]. Molecular modeling is an important technique for suggesting a rational to structure-activity relationship results, but the ability of computational approaches to predict affinity change is still challenging [151,152]. It is even more challenging to predict the relative affinity for different molecular targets, even if some success in this area were made using machine learning for predicting the type of voltage-gated ion channels targeted by some venom toxins [153,154,155,156]. A major challenge of molecular modeling and bioinformatics of toxins, such as snake toxins, is the prediction of their selectivity. Indeed, most of the toxins target ion channels that exist as multiple subtypes, the modulation of which have dramatically different consequences. It has been suggested that phenotypic screening approaches would have a better chance to discover drugs with a novel mode of action, than trying to modulate a particular molecular target, an approach that is currently preferred by pharmaceutical industries [4]. In this regard, bioinformatics could be used as a pre-screen to identify compounds that are more likely to have different activity than the already characterized toxins. This could, for example, be done using basic physicochemical characteristic predictions, sequence pattern recognition of activity at certain targets, or the prediction of structural motifs related to activity at molecular targets.

## Figures and Tables

**Table 1:** Snake venoms.

Snake Venom Protein Family	Entries	3D Structure		
		X-ray	NMR	Model
5′-nucleotidase	3			
AB hydrolase superfamily, lipase family	2			
AVIT (prokineticin)	1		1	
Bradykinin-potentiating peptide	65	2		
Cathelicidin	10		1	
Complement C3 homolog	5	5		
CRISP	66	9		
Crotamine-myotoxin *	17	1	2	
Cystatin	17			
Desintegrin *	33	5	5	
Endothelin/sarafotoxin	6		3	
Flavin monoamine oxidase (l-amino acid oxidase)	51	9		
Glycosyl hydrolase 56 (hyaluronidase)	9			
Multicopper oxidase	3	1		
Natriuretic peptide	55	2	1	
NGF-beta	38	1		
Nucleotide pyrophosphatase/phosphodiesterase	2			
Ohanin/vespryn	4			
PDGF/VEGF growth factor	20	2		
Peptidase S1 (serine protease)	202	11		4
Phospholipase A2	469	187		2
Phospholipase B-like	2			
pHpG (metalloprotease inhibitor) *	8			
Snaclec *	172	60		2
Snake three-finger toxin *	505	65	65	18
TCTP (translationally controlled tumor protein)	3			
True venom lectin (C-type lectin)	44	2		8
Type-B carboxylesterase/lipase	2	1		
Venom, Kunitz-type	143	8	6	
Venom metalloproteinase (M12B)	255	39	16	3
Not in a family	12			
Total	2224	410	100	37

* Those entries that are specific to venomous taxa are marked by an asterisk.
